# Antibodies to the RNA Binding Protein Heterogeneous Nuclear Ribonucleoprotein A1 Colocalize to Stress Granules Resulting in Altered RNA and Protein Levels in a Model of Neurodegeneration in Multiple Sclerosis

**DOI:** 10.4172/2155-9899.1000402

**Published:** 2016-03-22

**Authors:** Joshua N. Douglas, Lidia A. Gardner, Hannah E Salapa, Michael C. Levin

**Affiliations:** 1 Department of Neurology, University of Tennessee Health Science Center, Memphis, TN, 38104, USA; 2 Neuroscience Institute, University of Tennessee Health Science Center, Memphis, TN 38163, USA; 3 Research Service, VA Medical Center, Memphis, TN, 38104, USA

**Keywords:** RNA binding protein, Multiple sclerosis, hnRNP A1, Stress granules, Neurodegeneration, Spastin

## Abstract

**Objective:**

Multiple sclerosis (MS) is the most common demyelinating disorder of the central nervous system (CNS). Data suggest that antibodies to CNS targets contribute to the pathogenesis of MS. MS patients produce autoantibodies to heterogeneous nuclear ribonucleoprotein A1 (hnRNP A1). hnRNP A1 is an RNA binding protein (RBP) overexpressed in neurons that functions in pre-mRNA splicing, mRNA trafficking, and translation. Previously, we showed that anti-hnRNP A1 antibodies entered neuronal cells (*in vitro*) *via* clathrin-mediated endocytosis, caused mislocalization of endogenous hnRNP A1 protein and increased markers of neurodegeneration including decreased ATP concentration and apoptosis. In this study, we hypothesized that anti-hnRNP A1 antibodies might cause stress granule formation and altered levels of RNAs and proteins that bind hnRNP A1.

**Methods:**

Neuronal cell lines were exposed to anti-hnRNP A1 and isotype-matched control antibodies *in vitro* and examined for neuronal granule formation, including stress granules, P bodies and transport granules. In addition, RNAs that bound hnRNP A1 were determined. Levels of RNA and their translated proteins were measured upon exposure to the anti-hnRNP A1 antibodies.

**Results:**

Anti-hnRNP A1 antibodies induced and localized to stress granules, a marker of neurodegeneration, within a neuronal cell line. The anti-hnRNP A1 antibodies did not induce P bodies or neuronal granules. Clinically relevant RNAs were found to bind hnRNP A1. In addition, the anti-hnRNP A1 antibodies caused reduced levels of RNA and protein of the spinal paraplegia genes (SPGs) 4 and 7, which when mutated mimic progressive MS.

**Conclusions:**

Taken together, these data suggest potential mechanisms by which autoantibodies may contribute to neurodegeneration in MS.

## Introduction

Multiple sclerosis (MS) is an autoimmune disorder of the central nervous system (CNS) of unknown etiology. It is believed to occur in individuals with genetic susceptibility [1–5]. Disease in these individuals might be initiated by an environmental stimulus, which results in an inflammatory response directed towards CNS targets. The resulting autoimmune response leads to demyelination and axonal degeneration within the CNS.

MS patients have been shown to produce autoantibodies to various myelin and non-myelin antigens. For example, patients develop autoantibodies to myelin oligodendrocyte glycoprotein (MOG), myelin associated glycoprotein (MAG), myelin basic protein (MBP), and proteolipid protein (PLP) [6–9]. These myelin autoantibodies have been found in the cerebrospinal fluid (CSF) and serum of MS patients. Contradictory studies exist as to whether these myelin-associated antibodies are pathogenic [7,10–17]. In addition to myelin antigens, MS patients have been shown to produce autoantibodies to various neuronal antigens such as neurofascin (NF-186), neurofilament light chain (NF-L), and to glial antigens such as KIR4.1 and glial fibrillary acidic protein (GFAP) [18–21]. Importantly, studies have shown these target specific antibodies to be associated with increased disease progression in animal models (NF-186) and cerebrospinal fluid (CSF) from human samples (NF-L, GFAP) respectively [18–20].

Furthermore, in contrast to healthy controls and patients with Alzheimer’s disease, MS patients specifically produce autoantibodies to the non-myelin antigen heterogeneous nuclear ribonucleoprotein A1 (hnRNP A1) [22–27]. hnRNP A1 is an RNA binding protein (RBP) overexpressed in neurons [12,22,24–29]. hnRNP A1 contains two RNA binding motifs along its N-terminus as well as a glycine-rich, low-complexity C-terminal domain containing the “M9” sequence. ‘M9’ is the nuclear export sequence/nuclear localization sequence (NES/NLS) responsible for nuclear - cytoplasmic transport of hnRNP A1 [12,22,24–29]. Additionally, M9 binds transportin protein, which is necessary for nuclear - cytoplasmic transport of hnRNP A1 and mRNAs bound to it [12,24–29].

Autoantibodies produced by MS patients are specific to the M9 region of hnRNP A1 [12,22,24–27]. *In vitro*, anti-hnRNP A1-M9 antibodies were found to enter neuronal cells through a mechanism involving clathrin-mediated endocytosis [12]. Once inside neuronal cells, anti-hnRNP A1-M9 antibodies reduced cellular ATP levels, increased apoptosis, and caused endogenous hnRNP A1 mislocalization from a primarily nuclear localization to a near 1:1 nuclear to cytoplasmic ratio [12]. Furthermore, anti-hnRNP A1-M9 antibodies transfected into human neuronal cells in vitro altered RNA levels of the spastic paraplegia genes (SPGs) spastin (SPG4), spartin (SPG20), and paraplegin (SPG7) as shown by microarray [24]. The alterations in the SPGs were further confirmed in neurons purified from human brain samples of MS patients compared to normal controls by Real-Time PCR (RT-PCR) [24]. Mutations within the SPGs are associated with the neurologic disease hereditary spastic paraplegia, which is clinically similar to some forms of MS [30].

Three main types of granules exist within neuronal cells: stress granules (SGs), processing bodies (P bodies), and neuronal transport RNPs (also known as neuronal ‘transport granules’) [31]. SGs develop as the result of various cellular stressors [19]. SGs harbor translationally repressed RBPs and their mRNA cargo during cellular stress. Once the cell has returned to homeostasis; the SGs sort, remodel, and export the various mRNAs for reinitiation or storage [31]. P bodies act as sites of translational repression and degradation for the cell [31]. P bodies do not contain ribosomal subunits and are the waste management system of the cell. Neuronal transport granules act as motile granules to transport RBPs and their translationally arrested mRNA cargoes to sites of translation [31].

In summary, SGs harbor translationally arrested RBPs and their cargo during times of cellular stress until homeostasis is achieved, P bodies manage the waste of the cell, and neuronal transport granules are responsible for transport of RBPs and their translationally arrested cargoes, which upon arrival to designated sites in the neuron, undergo translation.

In this paper we examined whether anti-hnRNP A1-M9 antibodies interacted with the three types of neuronal granules (SGs, P bodies, and neuronal ‘transport granules’). Additionally, we tested whether the SPGs (SPG4, SPG7 and SPG20) are mRNA binding partners of hnRNP A1 protein. Furthermore, we determined the downstream effects of anti-hnRNP A1-M9 antibodies on translation of these clinically relevant mRNAs.

## Materials and Methods

### Cells

The SK-N-SH cell line (ATCC, HTB-11), an immortalized human neuroblastoma cell line was maintained in Dulbecco’s modified Eagle’s medium/F12 supplemented with 10% fetal bovine serum and 1% penicillin-streptomycin antibiotics. 100 mm plates were seeded for the RNA immunoprecipitation, RT-PCR, and Western blot experiments. 10^5^ SK-N-SH cells were seeded per well into 500 uL of complete DMEM/F12 media into 8 well chamber slides (Corning #354632) for RNA granule immunocytochemistry experiments.

### Exposure of SK-N-SH cells to antibodies *in vitro*

Anti-hnRNP A1 antibodies were obtained from Abcam (ab4791- rabbit polyclonal, ab5832 - mouse monoclonal) and Millipore (05-1521- mouse monoclonal; 04-1469 – mouse monoclonal). The anti-hnRNP A1 antibodies are specific for hnRNP A1-M9 and have been shown to overlap the M9 immunodominant epitope recognized by IgG isolated from MS patients [24]. Isotype-matched anti-rabbit IgGs were obtained from Abcam (ab107866) and Millipore (12-370). Antibodies were added to the culture media of SK-N-SH cells at a concentration of 8 ug/ml for each assay as determined in previous studies [12]. Primary antibodies utilized for protein detection by Western blot were as follows: spastin (SPG4-Abcam ab38150), paraplegin (SPG7-Abcam ab154989), spartin (SPG20-Abcam ab94950), and hnRNP A1-(Millipore 05-1521). Antibodies were used at manufacturers recommended concentration. Antibodies utilized for immunochemistry in granule studies were as follows: TAR-DNA Binding Protein (TDP-43 (Millipore MABN45)), GW-182 (Abcam ab70522), or hnRNP A2/B1 (Abcam ab6102).

### Immunocytochemistry of hnRNP A1-M9 antibody colocalization with neuronal granules

10^5^ SK-N-SH cells were seeded per well into 500 uL of complete DMEM/F12 media into 8 well chamber slides (Corning #354632). Anti-hnRNP A1 and control isotype-matched IgG2b antibodies were labeled with Alexafluor 488 and added to the culture media of SK-N-SH cells at a concentration of 8 ug/ml for each assay as determined in previous studies [12]. 48 hours following antibody addition, cells were fixed with 4% paraformaldehyde for 30 minutes at room temperature (RT). Fixed cells were then blocked and permeabilized with 6% Milk-PBS + 0.4% TritonX-100. Cells were then labeled for TDP-43 (stress granules), GW-182 (P bodies), or hnRNP A2/B1 (transport granules) at the manufacturer’s suggested concentration overnight at 4°C. Following primary antibody incubation, cells were washed 4 X 5 minutes with PBS. Cells were treated with secondary anti-mouse Cy3 at manufacturer’s suggested concentration for 2 hours at RT. Cells were washed 4 X 5 minutes with phosphate buffered saline (PBS), mounted with DAPI mounting medium, and imaged. Colocalization events were quantified as follows. Ten images were taken per treatment group (IgG and anti-hnRNP A1-M9 antibody addition). Within these images, cells were counted as well as the number of colocalizations of TDP-43 marked stress granules and antibody (IgG or anti-hnRNP A1-M9). Approximately 500 total cells were counted per group. The total number of colocalizations was compared to the total number of cells counted across the ten images. Data was analyzed by one-tail t-test based on previously observed trend [12]. (*p ≤0.05).

### RNA immunoprecipitation

SK-N-SH cells were cultured in 100 mm cell culture dishes. Cells were scraped and lysate extracted. 60 mcg of protein extracted from whole cell lysate was added to protein A/G beads labeled with either anti-hnRNP A1 antibody (ab4791) or anti-IgG (Millipore 12-370) as a control for non-specific binding. Samples were rotated overnight at 4°C. The following day, bound proteins were eluted from the protein A/G beads. Eluent from both samples as well as control SK-N-SH lysates were run on (sodium dodecyl sulfate) SDS gels and probed for hnRNP A1 to confirm specific binding of hnRNP A1 protein. RNA was then extracted from the eluents using the RNA-Stat 60 protocol [32]. RNA concentration and quality was determined by 260/230 ratio by Nanodrop (ThermoFisher Nanodrop 1000). RNA was used as a template for first strand synthesis to make cDNA using a High Capacity cDNA Reverse Transcription kit (Applied Biosystems 4368814) and a thermocycler. cDNA concentration and quality was determined by Nanodrop. cDNA was used to perform quantitative real-time PCR probing for potential RNA binding partners to hnRNP A1 protein.

### Determination of RNA levels of antibody treated SK-N-SH cells by quantitative RT-PCR

SK-N-SH cells seeded in 100 mm dishes were treated with either anti-hnRNP A1 antibodies, control anti-IgG antibodies (isotype matched), or were left untreated as previously described using protocols in our lab [12]. Antibody treatments were at a concentration of 8 ug/ml in DMEM/F12 media for 48 hours. Following antibody treatment, RNA was extracted and amplified as described.

### Determination of protein levels of antibody treated SK-N-SH cells by Western blot

SK-N-SH cells seeded in 100 mm dishes were treated with either anti-hnRNP A1 antibodies, anti-IgG antibodies, or were left untreated. Antibody treatments were at a concentration of 8 ug/ml in DMEM/F12 complete media for 48 hours. Following antibody treatment, cells were scraped and lysates were centrifuged 5 min at 2500 rpm. Supernatants were heated with 2X running buffer for 5 minutes at 95°C and then samples were loaded onto 10% sodium dodecyl sulfate polyacrylamide gel electrophoresis (SDS-PAGE) and run at 100V for 1.5 hours. Gels were then transferred onto nitrocellulose membranes at 100V for 1.5 hours. Membranes were blocked with 6% milk-TBS for 40 minutes at RT. Primary antibodies were then added overnight at 4°C at manufacturer’s suggested concentration. Primary antibodies were as follows: SPG4 (Abcam ab38150), SPG7 (Abcam ab154989), SPG20 (Abcam ab94950), hnRNP A1 (Millipore 05-1521), GAPDH (Abcam ab9485), and Beta-actin (Novus NB600-503). The next day, membranes were washed 4 X 5 min with Tris buffered saline (TBS). Secondary antibodies were then added at a 1:10,000 dilution (50 minutes, RT). Membranes were washed 4 X 5 minutes with TBS-Tween, developed and imaged. To compare relative changes in signal intensity for each protein tested, the following technique was utilized. Using Image J software, signal intensity was calculated for each Western blot. Next, percent signal intensity was calculated for each protein tested (Beta-actin, hnRNP A1, SPG 4, SPG 7 and SPG 20). For each protein, the relative signal intensity was calculated relative to the whole cell lysate signal. The adjusted signal intensity was normalized relative to the loading control (Beta-actin or GAPDH). Percent change of signal intensity of anti-hnRNP A1 antibodies was compared relative to control IgG for each protein tested (Supplementary file 1).

## Results

### Anti-hnRNP A1 antibodies colocalize with SGs but not P bodies or neuronal transport granules

**Figure 1:**
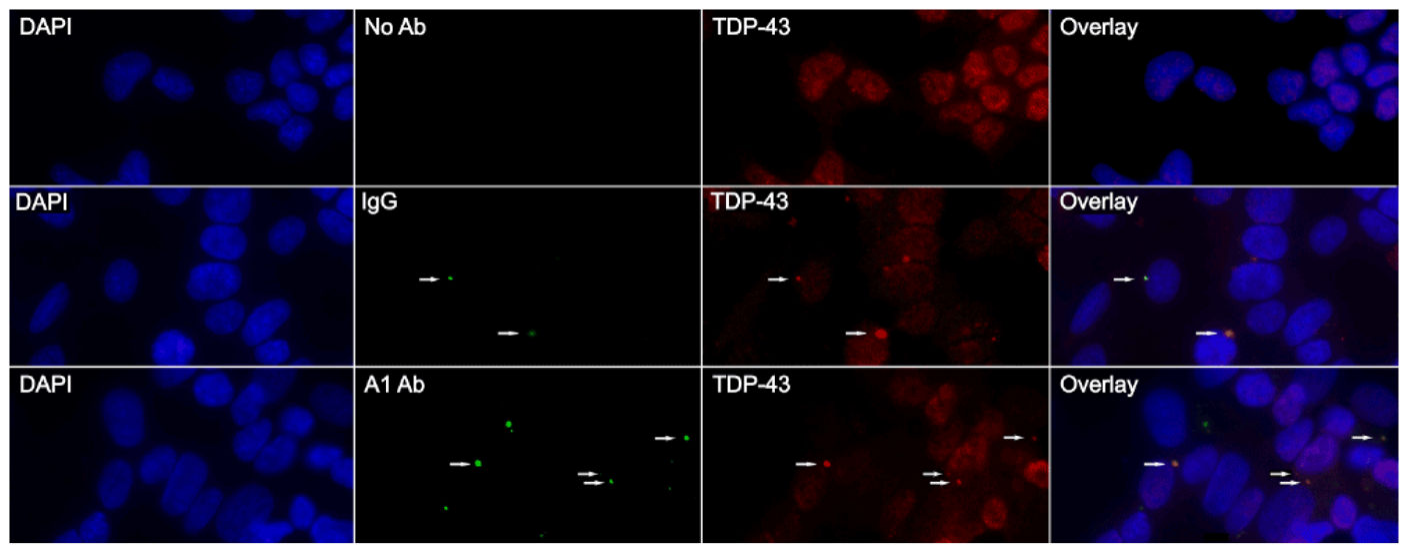
Antibodies co-localize with Stress Granules in SK-N-SH neurons. SK-N-SH cells were seeded into 8 chamber slides. Cells were treated with either anti-hnRNP A1-M9 antibodies, IgG, or left untouched. Following a 48 hr incubation, cells were fixed with 4% Paraformaldehyde, blocked and permeabilized, and stained for stress granules with anti-TDP-43 antibodies. The figure shows that both anti-hnRNP A1-M9 antibodies and control IgG (arrows) cause stress granule formation and colocalize to stress granules.

Neuronal granules play a crucial role in the trafficking of various RBPs (including hnRNP A1 [33]) and their mRNA cargoes. Since patients with MS produce antibodies to hnRNP A1, we hypothesized that anti-hnRNP A1-M9 antibodies would cause cellular stress resulting in SG formation at higher levels than control IgG. To answer this question, rabbit anti-hnRNP A1-M9 specific antibodies and isotype-matched rabbit control IgG labeled with Alexafluor 488 were added to the media of SK-N-SH neuronal cells in culture. After a 48 hour incubation, cells were fixed and labeled for SGs (TDP-43), P bodies (GW182), and neuronal transport granules (hnRNP A2B1) utilizing neuronal granule components previously identified by Kiebler et al. [31]. Our results revealed that both anti-hnRNP A1-M9 antibodies and control IgG resulted in SG formation and both colocalized with SGs (Figure 1). However, anti-hnRNP A1-M9 antibodies colocalized with SGs more readily and the differences were statistically significant (p≤0.05) (Table 1), suggesting that hnRNP A1-M9 antibodies specifically bound hnRNP A1 within SGs. Although absolute differences were small in the anti-hnRNP A1-M9 compared to isotype control antibody group, these differences were statistically significant and congruent with other studies [34]. Neither of the antibodies colocalized with P bodies or neuronal transport granules (not shown).

### SPG4 and SPG7 RNA bind hnRNP A1 protein

After determining that both anti-hnRNP A1-M9 antibodies and control IgG colocalize within SGs, we examined whether anti-hnRNP A1-M9 antibodies might have a specific effect on the RNA metabolism of hnRNP A1 protein’s RNA cargo. In order to accomplish this goal we needed to define the RNA binding partners of hnRNP A1 protein. To evaluate which RNAs might theoretically bind hnRNP A1, we used the RBPDB (RNA Binding Protein Data Base, http://rbpdb.ccbr.utoronto.ca/). This database contains the unique RNA binding sequences that are required for RNA to bind RNA binding domains of an RBP. The user enters an RNA sequence into the RBPDB. The RBPDB scans the RNA sequence and reports out all RBPs that might bind to it, and the exact sequence and its location within the RNA sequence input. We previously showed that anti-hnRNP A1 antibodies transfected into a neuronal cell line caused altered RNA levels of the SPGs (SPG 4, 7 and 20) [24]. Therefore, we hypothesized that these RNAs might be binding cargoes for hnRNP A1. Using the RBPDB, we found that spastin (SPG4) contains a 100% binding sequence (NM_014946, b.3283-3288) match with hnRNP A1’s RNA binding sequence in its non-coding sequence. SPG7 and SPG20 had variable degrees of RNA binding sequence alignment. Besides the RNA sequence, a number of variables contribute to RNA binding, such as the ability for RNAs to bind multiple RBPs, the quantity of the RBP present in the cells being analyzed and the strength of the binding between the RNA and the RBP [35]. Thus, RNA binding to hnRNP A1 is not the only mechanism by which RNAs might be altered in this system. Each RNA has to be examined experimentally, which we did in the following experiments. hnRNP A1 RNA was also tested because it previously was shown to be an RNA binding partner of hnRNP A1 as determined by CLIP analysis [36].

**Figure 2:**
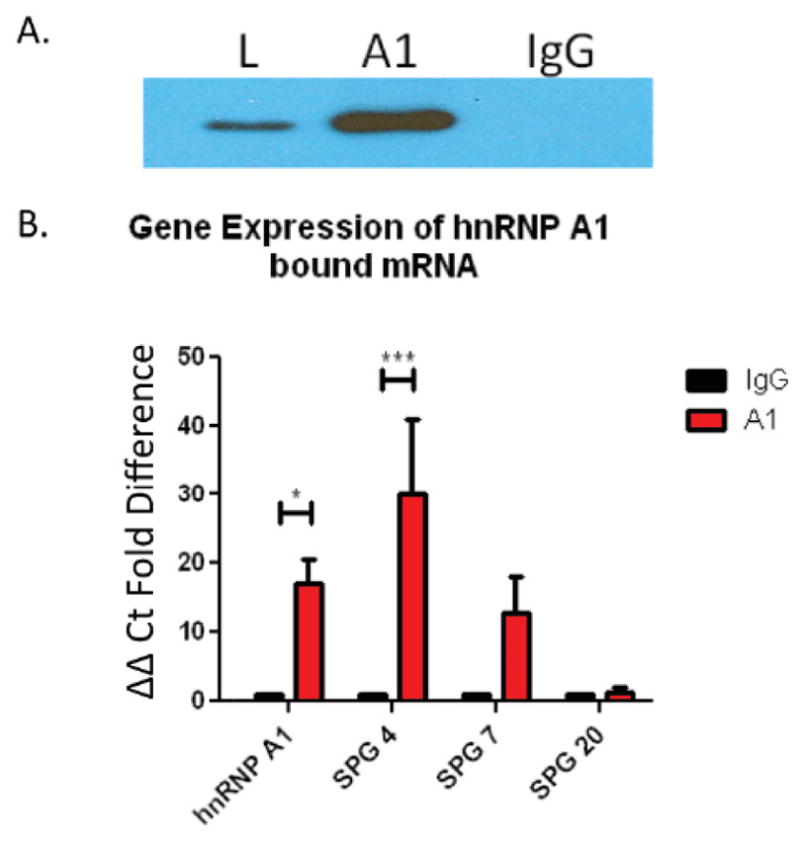
RNA Immunoprecipitation (IP). SK-N-SH cells lysates were incubated with protein A/G agarose beads labeled with antibodies to either hnRNP A1 (ab4791) or IgG (Millipore 12-370). Proteins and any bound RNA partners were eluted from the beads. (A) Protein eluents were run on 10% Tris-glycine gels for Western blotting, probed for hnRNP A1 protein (Millipore 4B10 05-1521). L=Untouched SK-N-SH lysate, A1=hnRNP A1 bound agarose beads eluent, IgG=IgG bound agarose beads eluent. The blots show that hnRNP A1 was present in the lysate (L) and the anti-hnRNP A1 IP (A1) but not in the control IgG IP (IgG). (B) RNA was isolated from protein eluent for each group and amplified by RT-PCR (see methods). Values within groups were normalized to GAPDH. Results revealed that hnRNP A1 and SPG4 are RNA binding partners of hnRNP A1 and SPG-7 showed a positive trend towards RNA binding. ^*^p ≤ 0.05 analyzed by t-test

To test whether the target RNAs bound hnRNP A1, we performed RNA immunoprecipitation (IP), followed by RT-PCR of the resulting eluent. Specifically, protein A/G agarose beads were labeled with anti-hnRNP A1-M9 antibodies to elute hnRNP A1 protein with its bound RNA. In parallel, protein A/G agarose beads were labeled with isotype-matched IgG (control). SK-N-SH lysates were incubated with the antibody-bound beads, bound protein-RNA complexes were eluted and the eluents run on Western blot (Figure 2A). Following IP with anti-hnRNP A1-M9 antibodies, a positive signal was detected, indicative of anti-hnRNP A1 antibody – hnRNP A1 protein interaction (a successful IP). Under identical conditions, the isotype control IgG did not bind hnRNP A1, thus control IgG did not bind hnRNP A1 protein and therefore there was no signal (Figure 2A).

After determining the specificity of the immunoprecipitation, RNA was extracted from the eluents using the RNA-Stat 60 protocol [32]. Following first strand synthesis, cDNA was used for quantitative RT-PCR to determine if the target RNAs chosen from the microarray data were present, indicative of their binding to hnRNP A1 protein. Glyceraldehyde 3-phosphate dehydrogenase (GAPDH) was used as a control to normalize each group. Our results revealed that hnRNP A1, SPG4 and to a lesser extent, SPG7 bound hnRNP A1 (Figure 2B). In contrast, SPG20 did not bind to hnRNP A1 (Figure 2B).

### anti-hnRNP A1-M9 antibodies alter SPG4 and SPG7 RNA levels by RT-PCR

**Figure 3:**
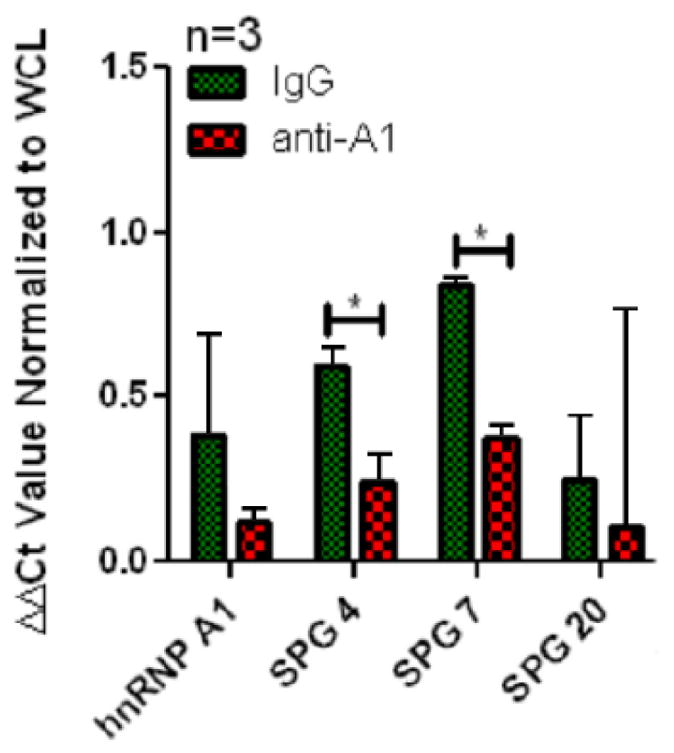
Anti-hnRNP A1 antibodies alter RNA levels as measured by RT-PCR. SK-N-SH cells were cultured in 100mm plastic dishes. Cells were treated with anti-hnRNP A1-M9 antibodies or isoytpe-mathced control IgG at a concentration of 8ug/mL. Following a 48 hour incubation, cells were lysed and RNA extracted. RNA quality was determined by nano-drop and cDNA was synthesized. RT-PCR was run in duplicates to measure RNA levels of genes of interest and each experiment was performed three times. Results (mean ± standard deviation) revealed decreased levels of SPG4 and SPG7 RNA levels in anti-hnRNP A1-M9 antibody treated cells compared to controls. ^*^p ≤ 0.05 analyzed by t-test. WCL: Whole Cell Lysate.

After determining that SPG4 and SPG7 were RNA binding partners of hnRNP A1 protein, we tested whether the addition of anti-hnRNP A1-M9 antibodies altered RNA levels of these genes. We hypothesized that anti-hnRNP A1-M9 antibodies would alter RNA levels of hnRNP A1’s binding partners (SPG 4, SPG 7 and hnRNP A1) compared to RNA that did not bind hnRNP A1 (SPG 20). We performed these studies by adding 8 ug/ml of anti-hnRNP A1-M9 antibodies or control IgG to SK-N-SH neuronal cells. Cells were incubated with antibodies for 48 hours, lysed and RNA extracted and amplified as described previously. Values obtained from the anti-hnRNP A1-M9 treated cells were compared to the control IgG treated cells to determine the effect on RNA expression due to the specificity of the anti-hnRNP A1-M9 antibodies (Figure 3). The results of these experiments indicate that RNA levels of SPG4 and SPG7 were altered compared to controls (Figure 3).

### anti-hnRNP A1-M9 antibodies alter SPG4 and SPG7 protein levels by Western blot

**Figure 4:**
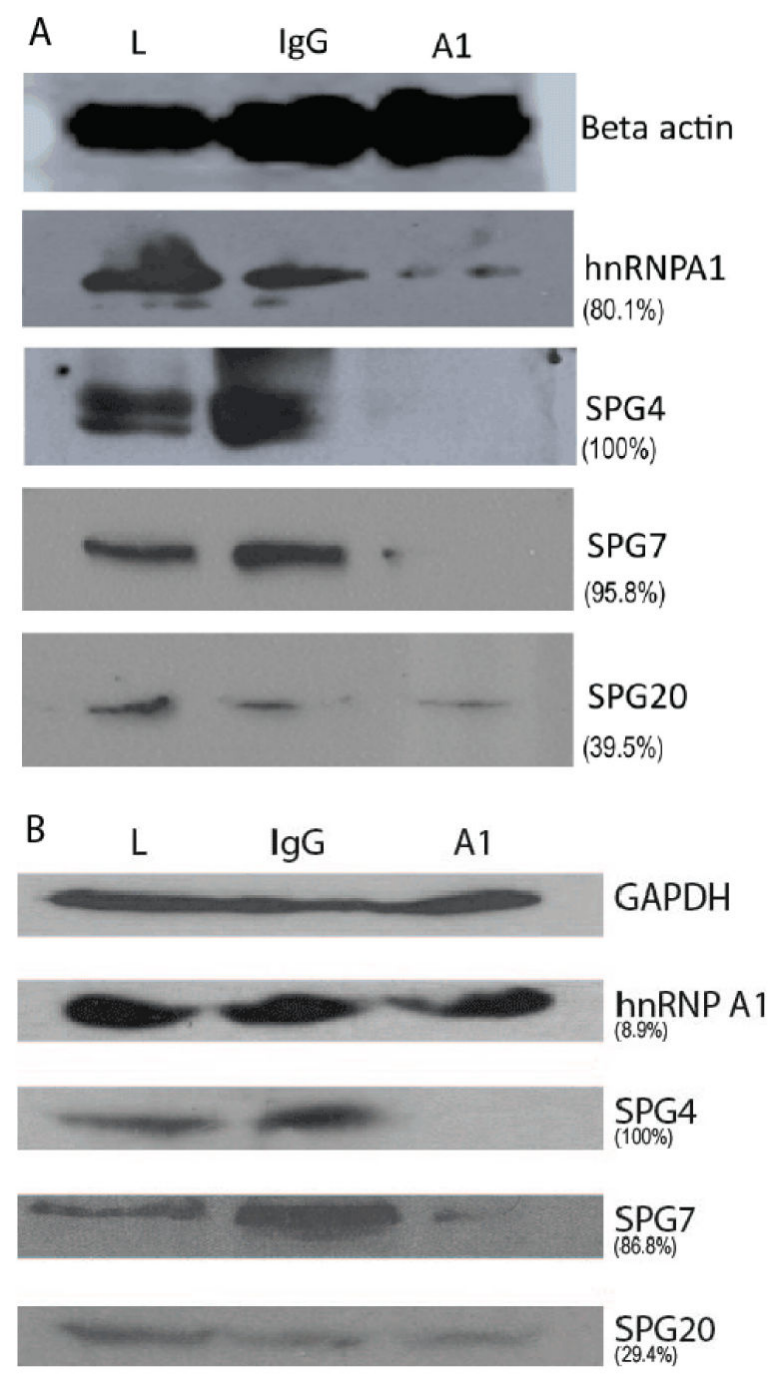
Anti-hnRNP A1 antibodies alter protein levels as measured by Western blot. SK-N-SH cells were cultured and treated with anti-hnRNP A1 antibodies or control IgG. Following a 48 hour incubation, cells were lysed and protein lysate was run on 10% Tris-glycine gels for Western blot analysis and probed for Beta-actin (A) or GAPDH (B)) (control), hnRNP A1, SPG4, SPG7, and SPG20. Results revealed that there was a marked reduction of SPG 4 and SPG 7 protein levels in anti-hnRNP A1 antibody compared to control isotype IgG treated cells. There was a variable response to hnRNP A1 protein and a modest reduction of SPG 20. Parentheses show relative percent reduction of signal comparing anti-hnRNP A1 antibody to control isotype IgG treatment of cells. (L=lysate, IgG=control isotype IgG, A1=anti-hnRNP A1-M9 antibody treatment of cells).

Next, we hypothesized that protein levels of hnRNP A1’s RNA binding partners (hnRNP A1, SPG4 and SPG7) would be altered whereas RNAs that did not bind hnRNP A1 (SPG-20) would not. Additionally, since anti-hnRNP A1-M9 antibodies and isotype control IgG both initiated SG formation and colocalized within SGs, we wanted to determine if anti-hnRNP A1-M9 antibodies caused specific downstream effects (i.e. altered translation) upon the bound RNAs compared to isotype control IgG. We performed these studies by adding 8 ug/ml of anti-hnRNP A1-M9 antibodies or control IgG to SK-N-SH neuronal cells. These data were compared to untreated SKN-SH neuronal cells run in parallel. Cells were incubated with antibodies for 48 hours, lysed and proteins extracted for Western blotting. We found dramatic differences in protein expression of SGP4 (100% in both experiments) and SPG 7 (experiment 1: 95.8%, experiment 2: 86.8%) in the anti-hnRNP A1-M9 antibody compared to isotype control treated cells (Figure 4, Supplementary file 1). Differences were variable for hnRNP A1 and only modest for SPG 20 (experiment 1: 39.5%, experiment 2: 29.4%). Taken together, these data indicate that in contrast to isotype control IgG (which also localized to SGs), anti-hnRNP A1-M9 antibodies altered the translation of the RNAs that bind hnRNP A1.

## Discussion

Previous studies have shown that in contrast healthy controls as well as patients with other neurologic disease, MS patients develop antibodies to hnRNP A1 [12,22,24–26,28]. hnRNP A1 is a RBP highly expressed in neurons responsible for trafficking of mRNA from the nucleus to the cytoplasm for translation. hnRNP A1 has a C-terminal region termed ‘M9’, which is its NES/NLS responsible for the nuclear - cytosolic trafficking of hnRNP A1 [12,22,24–29]. The antibodies produced by MS patients are specific to this M9 region [12,22,24–27]. Using monoclonal antibodies that overlap the human immunodominant epitope of hnRNP A1–M9 recognized by IgG isolated from MS patients, we previously showed that these antibodies caused neurodegeneration, decreased cellular ATP levels and increased apoptosis in neuronal cell lines [12,24]. Consistent with the specificity of the anti-hnRNP A1-M9 antibodies for the M9 NES/NLS, we also showed that endogenous hnRNP A1 was redistributed from a primarily nuclear localization to an equal nuclear/cytoplasmic location [12]. Using these same antibodies, the current studies showed that anti-hnRNP A1-M9 antibodies caused cellular stress resulting in formation of SGs to which the antibodies colocalize. Additionally, we demonstrated that SPG4 and SPG7 are RNA binding partners of hnRNP A1. Furthermore, we determined that anti-hnRNP A1-M9 specific antibodies have profound effects upon specific RNA binding partners of hnRNP A1 protein. Specifically, we showed that SPG4 and SPG7 RNA and protein expression are reduced, which may be due to the specificity of anti-hnRNP A1-M9 antibodies compared to isotype control IgG.

Taken together, these studies suggest a potential mechanism by which autoantibodies might contribute to neurodegeneration. One of hnRNP A1’s many roles in RNA metabolism (‘ribostasis’) is to bind RNA, transport RNA from the nucleus to the cytoplasm, where it undergoes translation [37]. hnRNP A1 is then quickly transported back to the nucleus. Thus, under normal cellular homeostasis, hnRNP A1 is predominantly localized to the nucleus. Although further studies are warranted, these data suggest a series of events by which anti-hnRNP A1-M9 antibodies might alter hnRNP A1 function within neurons. First, anti-hnRNP A1-M9 antibodies enter neurons by clathrin-mediated endocytosis [12]. Second, the anti-hnRNP A1-M9 antibodies bind M9, which is hnRNP A1’s nucleocytoplasmic shuttling domain. The binding interrupts the normal transport of hnRNP A1 into the nucleus, thus hnRNP A1 is mislocalized to the cytoplasm, which might then cause cellular stress and SG formation. Interestingly, previous studies have shown that hnRNP A1 is a component of SGs when cells are exposed to either osmotic or oxidative stress [38,39]. The anti-hnRNP A1-M9 antibodies localize to SGs, which results in decreased levels of SPG4 and SPG7 mRNA and protein, two clinically relevant targets related to MS, which now have been shown to bind hnRNP A1. The decreased levels of target RNAs may subsequently contribute to neurodegeneration [12,22,24–27].

It is important to note that mutations in the genes affected (SPG4, SPG7) result in various forms of hereditary spastic paraplegia (HSP) [30]. HSP is a genetically inherited spastic disorder similar clinically to MS [30]. Mutations in SPG4 account for the most common autosomal dominant form of HSP [30]. Additionally, SPG7 is a gene associated with mitochondrial function, specifically with mitochondrial respiratory chain complexes I and IV [40]. It was shown that patients with HSP resulting from mutations in SPG7 showed a significant decrease in mitochondrial respiratory chain complexes I and IV [40]. Previously we have shown depleted ATP levels *in vitro* due to the addition of anti-hnRNP A1-M9 antibodies. In this article we show that anti-hnRNP A1-M9 antibodies alter protein levels of SPG7 *in vitro*. In concurrence with the studies in HSP patients with depleted mitochondrial respiratory chain complexes, our data suggests that altered SPG7 levels due to anti-hnRNP A1-M9 antibodies might act in a similar mechanism to the SPG7 mutations seen in patients with autosomal recessive HSP.

In summary, we showed that anti-hnRNP A1-M9 antibodies cause cellular stress resulting in SGs to which the antibodies colocalize. Additionally, we determined that SPG4, and SPG7 are RNA binding partners of hnRNP A1, and that addition of anti-hnRNP A1 antibodies to a neuronal cell line resulted in decreased levels of both SPG4 and SPG7 RNA and protein. The exact mechanism by which this occurs requires further study. For example, although we showed decreased levels of target proteins by Western blotting, it is not yet know whether this is due to changes in protein translation, degradation or altered RNA levels. Alternatively, the changes we observed might also be due to changes in hnRNP A1 protein (Figure 4), which might also alter levels of RNAs that bind hnRNP A1. Further, these results might be explained by impaired SG disassembly triggered by antibody binding, which in turn could also alter RNA metabolism [37]. Interestingly, M9 (which was targeted by antibodies in this study) is contained within the low complexity domain of hnRNP A1, which contributes to SG disassembly [37]. Although *in vivo* studies are required to clarify the role of anti-hnRNP A1-M9 antibodies in the pathogenesis of neurodegeneration in MS, an important link is made because (1) the monoclonal antibodies used in this study overlap the human immunodominant epitope of hnRNP A1–M9 recognized by IgG isolated from MS patients, (2) the SPG data supports previous data shown *in vitro* and in neurons isolated from MS brains and (3) the SPGs are clinically relevant targets [12,24]. Taken together, these studies emphasize the importance of autoantibodies to non-myelin antigens in the pathogenesis of MS and shed insight into a possible mechanism of how autoantibodies to hnRNP A1-M9 cause changes in neuronal function which ultimately leads to neurodegeneration.

## Supplementary Material

Supplementary file

## Figures and Tables

**Table 1:** Quantification of Antibody Colocalization within Stress Granules. Approximately 500 cells were imaged and the number of antibodies that colocalized to stress granules was counted (see Methods). The increase in colocalization was quantified using a one-tailed t-test based on a previously observed trend.

	IgG control	Anti-A1
Cells counted	537	530
Colocalizations	19	30
Average ± SD colocalizations per image (n=10 images/group)	1.9 ± 1.101	3.0 ± 1.491*
% cells containing colocalizations	3.54%	5.66%

* p≤0.05.
